# agReg-SNPdb: A Database of Regulatory SNPs for Agricultural Animal Species

**DOI:** 10.3390/biology10080790

**Published:** 2021-08-17

**Authors:** Selina Klees, Felix Heinrich, Armin Otto Schmitt, Mehmet Gültas

**Affiliations:** 1 Breeding Informatics Group, Department of Animal Sciences, Georg-August University, Margarethe von Wrangell-Weg 7, 37075 Göttingen, Germany; felix.heinrich@uni-goettingen.de (F.H.); armin.schmitt@uni-goettingen.de (A.O.S.); 2 Center for Integrated Breeding Research (CiBreed), Georg-August University, Albrecht-Thaer-Weg 3, 37075 Göttingen, Germany; 3 Faculty of Agriculture, South Westphalia University of Applied Sciences, Lübecker Ring 2, 59494 Soest, Germany

**Keywords:** single nucleotide polymorphism, regulatory SNP, transcription factor, transcription factor binding site, gene regulation, database, agricultural animal species, livestock

## Abstract

**Simple Summary:**

Regulatory SNPs (rSNPs) are SNPs located within promoter regions that have a high potential to alter gene expression by changing the binding affinity of transcription factors to their binding sites. Such rSNPs are gaining importance in the life sciences due to their causality for specific traits and diseases. In this study, we present agReg-SNPdb, the first database comprising rSNP data of seven agricultural and domestic animal species: cattle, pig, chicken, sheep, horse, goat, and dog, and made it usable via a web interface.

**Abstract:**

Transcription factors (TFs) govern transcriptional gene regulation by specifically binding to short DNA motifs, known as transcription factor binding sites (TFBSs), in regulatory regions, such as promoters. Today, it is well known that single nucleotide polymorphisms (SNPs) in TFBSs can dramatically affect the level of gene expression, since they can cause a change in the binding affinity of TFs. Such SNPs, referred to as regulatory SNPs (rSNPs), have gained attention in the life sciences due to their causality for specific traits or diseases. In this study, we present agReg-SNPdb, a database comprising rSNP data of seven agricultural and domestic animal species: cattle, pig, chicken, sheep, horse, goat, and dog. To identify the rSNPs, we constructed a bioinformatics pipeline and identified a total of 10,623,512 rSNPs, which are located within TFBSs and affect the binding affinity of putative TFs. Altogether, we implemented the first systematic analysis of SNPs in promoter regions and their impact on the binding affinity of TFs for livestock and made it usable via a web interface.

## 1. Introduction

The transcriptional regulation of gene expression in higher organisms is essential for various biological processes. In contrast to the process of translation, the transcriptional machinery and its regulatory mechanisms are far from being deciphered [1]. These mechanisms are mainly governed by a special class of regulatory proteins, the transcription factors (TFs), and their combinatorial interplay [2,3]. TFs regulate the transcription as a response to specific environmental conditions by binding to short degenerate sequence motifs known as transcription factor binding sites (TFBSs) in promoter regions of their target genes and, thereby, enhance or repress gene transcription. Genomic variations, such as single nucleotide polymorphisms (SNPs), define and characterize specific populations or phenotypes and are, hence, used as markers in animal and plant breeding.

**Figure 1:**
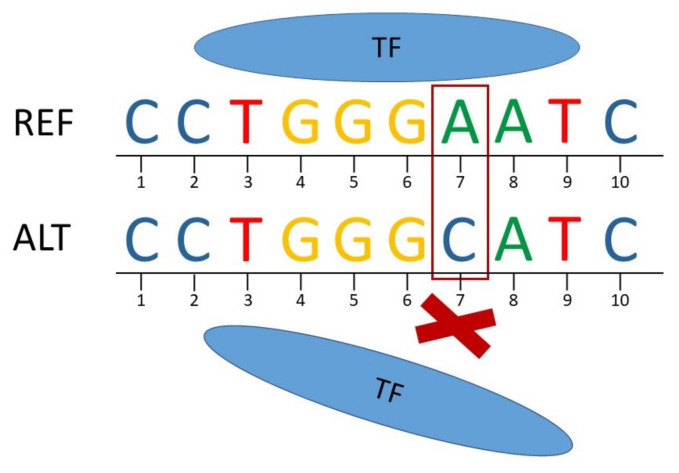
Scheme of the disruption of transcription factor (TF) binding due to a regulatory SNP. The TF can bind to the reference (REF) sequence while it does not bind to the alternate (ALT) sequence (C instead of A at position 7).

Due to the decreasing costs for whole genome sequencing, an increasing number of variants is detected followed by association studies statistically linking SNPs to specific traits or diseases. However, the identification of causal variants and the elucidation of their regulatory roles is proceeding at a slow rate [4,5]. Today, it is well known that most disease- and trait-associated SNPs are not located within the coding regions of genes but in non-coding regions [6,7,8,9]. SNPs that are located in regulatory regions can alter TFBSs leading to a change in the binding affinity of TFs and, in extreme cases, even result in the disruption of a TFBS or the creation of a new TFBS (Figure 1) and, thus, affect gene expression. Such SNPs are referred to as regulatory SNPs (rSNPs) [10,11,12].

The importance of rSNPs has been studied extensively in humans and they are found to have a causal role for numerous traits and diseases [13,14,15,16]. A recent review on human rSNPs summarizes different rSNP studies [6]. Due to the great interest in rSNPs, several tools and databases for the analysis of the effects of SNPs on regulatory elements, e.g., TFBSs, have been developed for humans or certain model organisms. Five recent studies are summarized in Table 1, and a comprehensive overview is given in Table S1.

Recently, rSNPs are gaining attention in life sciences and animal breeding since they can be causal for specific traits and diseases and could, hence, serve as new targets for breeding. For this reason, several studies investigated the critical role of rSNPs in agriculturally important species, such as cattle [17,18,19,20,21,22,23], pig [24,25,26], and chicken [27,28,29]. As these studies were focused on the regulatory role of SNPs for a single trait of interest, they were highly case-specific. Thus, there still exists a lack of systematic analyses of the effects of rSNPs in agricultural species, and, until now, only a few existing tools and databases (DBs) are available for livestock.

MotifbreakR [30] and atSNP [11] are both R packages that principally include all organisms stored in the Bioconductor BSGenome package [31]; however, they require the user to supply the SNP and TFBS data (represented by position weight matrices (PWMs)), and experience in R programming is essential. The Ensembl Variant Effect Predictor (VEP) [32] stores data from experimentally supported and published rSNPs. Due to the lack of experimentally supported data of regulatory elements in livestock, the VEP mainly contains data of regulatory elements and variants for human and mouse. Therefore, the information for livestock stored in the Ensembl VEP is limited to annotations based on the position of the SNP with respect to a gene, e.g., in the upstream region or in the 5′ UTR, excluding effects on TF binding.

**Figure 2:**
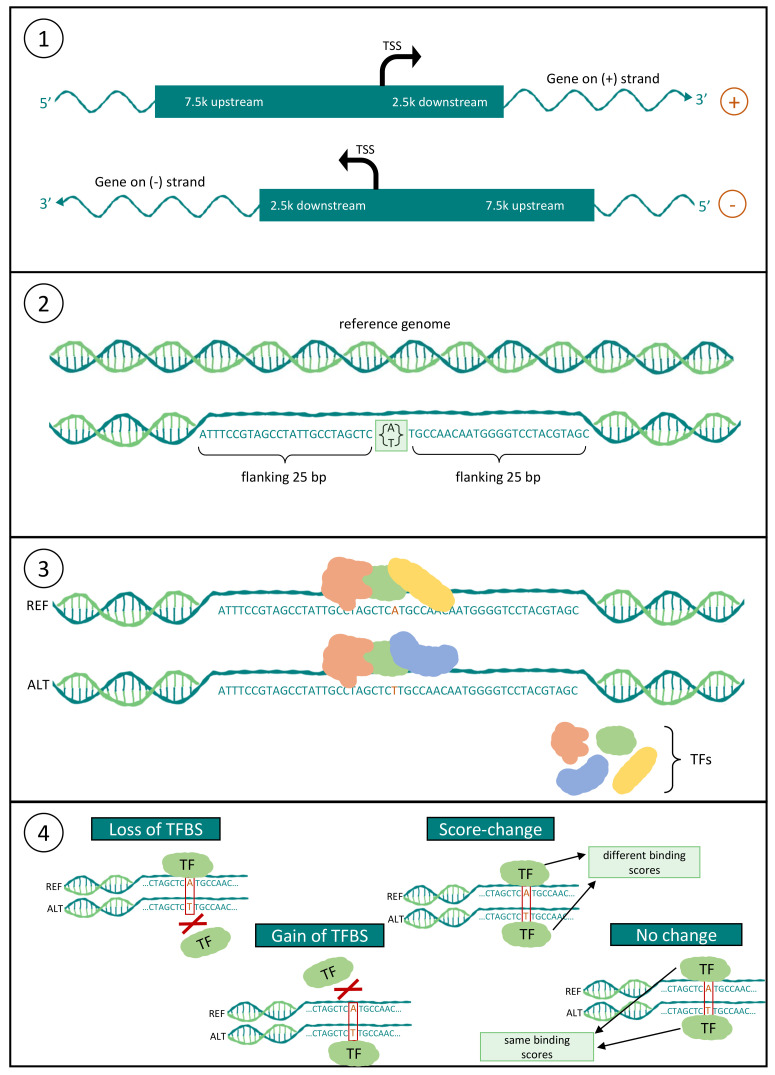
Scheme of the workflow applied for the detection of rSNPs. (**1**) Definition of the promoter region as 7.5 kb upstream (5′ direction) and 2.5 kb downstream (3′ direction) of the TSS, and extraction of SNPs within this region; (**2**) extraction of the flanking 25 bp around the SNPs from the reference genome; (**3**) prediction of the TFBSs for both the reference and alternate sequences; and (**4**) deriving the consequences for each SNP-TFBS pair.

In order to address the limited knowledge and information available regarding the crucial functions of rSNPs and their associations with TFBSs in livestock, we systematically carried out an analysis to detect rSNPs and predicted their effects on TF binding for seven agricultural and domestic species (cattle, pig, chicken, sheep, horse, goat, and dog). In particular, we first analyzed the promoter regions (ranging from −7.5 kb to +2.5 kb) of all annotated genes and obtained the SNPs within these regions. Secondly, we extracted the flanking sequences for these SNPs and performed a TFBS prediction on the reference as well as alternate sequences. Finally, we assigned the identified SNPs to different categories based on their consequences on TF binding (Figure 2) as suggested in [33,34]. To demonstrate our results in a proper way, we developed a database, namely agReg-SNPdb, which stores all predicted regulatory SNPs and their consequences on TF binding for each gene, and we made it accessible via a web interface (https://azifi.tz.agrar.uni-goettingen.de/agreg-snpdb, (accessed on 16 August 2021)). Furthermore, we performed a literature survey to show that our results are in agreement with previous experimental and in silico studies.

## 2. Materials and Methods

### 2.1. Input Data

The construction of agReg-SNPdb requires: (i) a library of PWMs representing the TFBSs and, for each animal, (ii) a reference genome, (iii) a SNP catalog, and (iv) gene annotations. As a PWM library, we used the non-redundant vertebrate matrices provided by TRANSFAC [40]. The reference genomes, SNP catalogs, and gene annotation files are downloaded from Ensembl [41]. The respective assembly versions are listed in Table 2. The SNP catalog was filtered by discarding all insertions and deletions, keeping only the SNPs. For most genes, more than one transcript isoform was annotated [32], e.g., due to different splicing variants. This ambiguity was kept during the analysis if the positions of the transcription start sites (TSSs) and, hence, the derived promoter regions were different.

### 2.2. Pipeline

A general workflow of the detection pipeline is shown in Figure 2. In our previous studies on faba beans [34] and rapeseed [33], we established similar pipelines for the prediction of rSNPs.

#### 2.2.1. Detection of SNPs within the Promoter Region

The first step of this analysis was to extract SNPs, which are located within the pre-defined promoter regions. Since there exists no experimentally verified information regarding the exact location of the promoters and in order to overcome inaccuracies in TSS prediction, we chose a large promoter region of 7.5 kb upstream and 2.5 kb downstream of the TSS. Similarly large promoter regions were used in previous studies [10,37,42,43,44,45,46,47,48]. This promoter region can be narrowed by the user during a database search on our website. For all annotated genes, we extracted the SNPs within this region for further analysis by using the function foverlaps of the package data.table in R [49].

#### 2.2.2. Prediction of TFBSs

For each SNP lying within a promoter region, we extracted the respective flanking sequence of 25 bp on each side of the SNP resulting in sequences with a total length of 51 bp and the SNP at position 26 (similar flanking sequences were used in [33,34,43,50]). Sequences with a length of less than 51 bp or sequences with gaps were discarded. After extracting the flanking sequences, we created two sequences per SNP, one with the reference and one with the alternate allele at the SNP position. Both were used as input for the TFBS prediction tool MATCH™ [51], which scanned the sequences to predict TFBSs using a PWM library from TRANSFAC with specific cutoff values to minimize the false positive rates. If a PWM matched a segment of genomic DNA, this sequence motif was referred to as a (potential) TFBS. As a result, the algorithm provided two scores for each predicted TFBS [40,51]: the matrix similarity score (MSS), measuring the quality of the match regarding the whole PWM sequence, and the core similarity score (CSS), measuring the quality of the match regarding the first five most-conserved consecutive positions of the PWM. Both scores were within the range [0, 1], where a score of 1 denoted an exact match of the sequence with the PWM [51] measuring the quality of the match and indicating the binding affinity of a TF to the site.

In TRANSFAC, a PWM identifier follows a certain terminology with the structure V$*factorname_version*. In our case, each PWM starts with “V$”, which indicates that the PWM originated from a vertebrate TF. The *factorname* specifies the name of the TF that is binding to the DNA motif. Since there can be several PWMs representing the sequence motif of a specific TF, the *version* was specified for unique identification [3,40].

#### 2.2.3. Annotation of Consequences

For each SNP, we obtained two sets of predicted TFBSs—one for the reference and one for the alternate allele. By comparing these two sets, we manually determined the consequence of a SNP on a TFBS as in our previous studies [33,34]. We differentiated four different consequences: (i) no effect, (ii) change in binding affinity, (iii) loss of TFBS, and (iv) gain of TFBS. We defined two TFBS predictions as the same if their PWMs, positions, and the strand on which they were found were equal for both alleles.

A SNP was considered to have no effect on a TFBS if both scores computed by MATCH™ were equal for both alleles. A SNP was considered to cause a change in the binding affinity of a TF if the matrix similarity score computed by MATCH™ differed for the two alleles. A SNP caused a loss or gain of TFBS if the considered TFBS was only predicted for the reference or alternate sequence, respectively. In this study, we defined an rSNP as a SNP that caused a loss or gain of TFBS or a score-change for at least one TFBS.

## 3. Results

### 3.1. Database

We created the mysql database [52] agReg-SNPdb, which stores (i) general information about the SNPs, such as the ID, chromosomal position and the alleles (table *snp_info*); (ii) general information about the genes, such as the gene name and chromosomal position (table *gene_info*); (iii) the table *snp_region* connecting the tables *snp_info* and *gene_info* by storing SNPs and their corresponding target genes together with their genomic position within the promoter region based on the distance to the TSS; and, most importantly, (iv) for each SNP within a promoter region (i.e., for each SNP in table *snp_region*), we store its consequences based on the predicted TFBS binding potential (table *TFBS_results*). A summary of the number of entries for each table and animal stored in our database is shown in Table 3.

### 3.2. Web Interface

**Figure 3:**
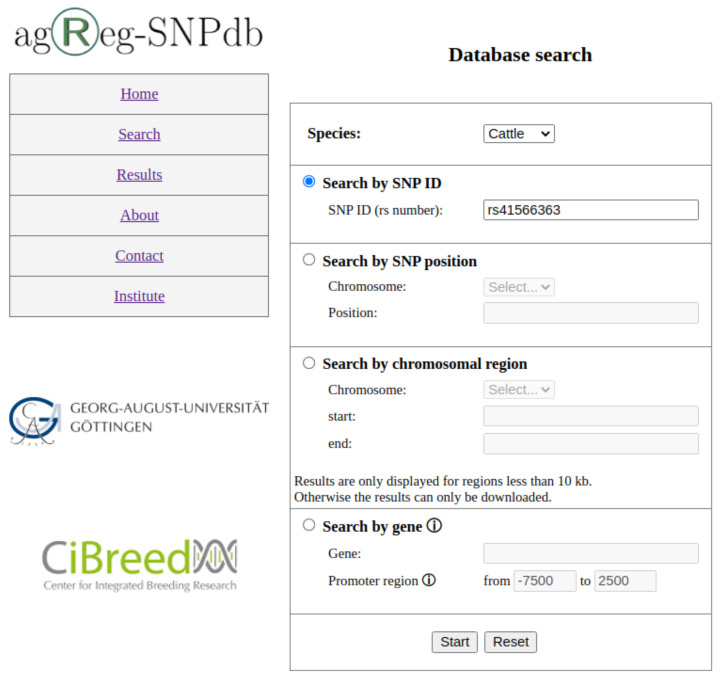
Search page of agReg-SNPdb. Search options are (1) by SNP ID, (2) by SNP position, (3) by chromosomal region, and (4) by gene.

The web interface (https://azifi.tz.agrar.uni-goettingen.de/agreg-snpdb, accessed on 16 August 2021) allows users to query the agReg-SNPdb without SQL knowledge and to obtain the requested results either on our website directly or by downloading them as CSV files. The database can be searched by (i) SNP identifiers in the form of rs numbers, (ii) SNP positions, (iii) SNP regions in a specified chromosome, or (iv) gene identifiers, i.e., the Ensembl gene stable ID or gene name (Figure 3).

**Figure 4:**
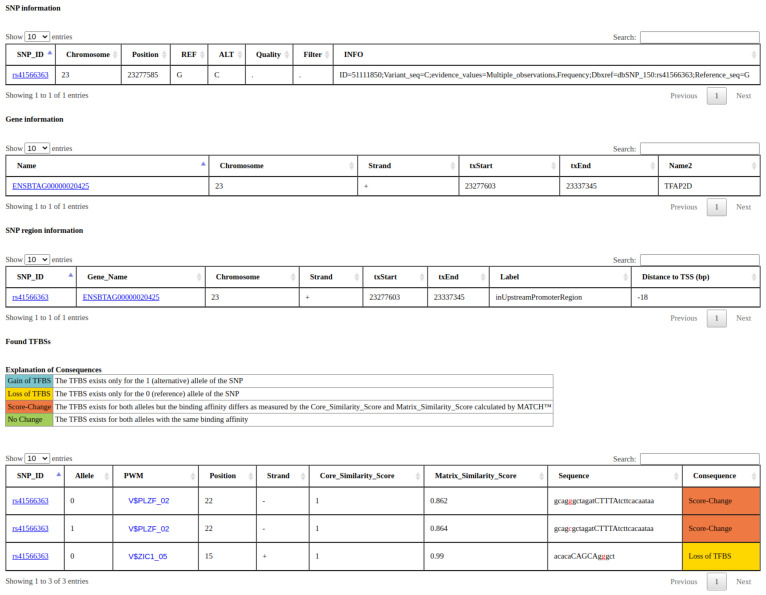
Example of a search result from agReg-SNPdb. The search was performed by the SNP id rs41566363 of cattle. The result tables contain, first, general SNP information; secondly, general gene information; thirdly, information about the SNP region, in particular the promoter region and distance to the TSS; and lastly, the overlapping TFBSs (represented by PWMs) for the SNP with predicted consequences.

The search results will contain, at maximum, four tables: (1) a table showing general SNP information (table *snp_info*); (2) a table showing general gene information (table *gene_info*); (3) a table linking the SNPs to the genes, more specifically to the promoter regions, if they are positioned within a promoter region (table *snp_region*); and (4) for all rSNPs, a table with the predicted TFBSs overlapping each rSNP, the MATCH™ scores, and the respective consequence (table *TFBS_results*) for both alleles. An example output can be seen in Figure 4. In all tables, we provide links to sites with additional information for the SNPs and genes, and, for each PWM, we display the respective sequence logo if desired. Apart from the search site, the complete database tables can be downloaded chromosome-wise on the summary page of the respective animal.

### 3.3. Statistical Analysis of the Data

**Figure 5:**
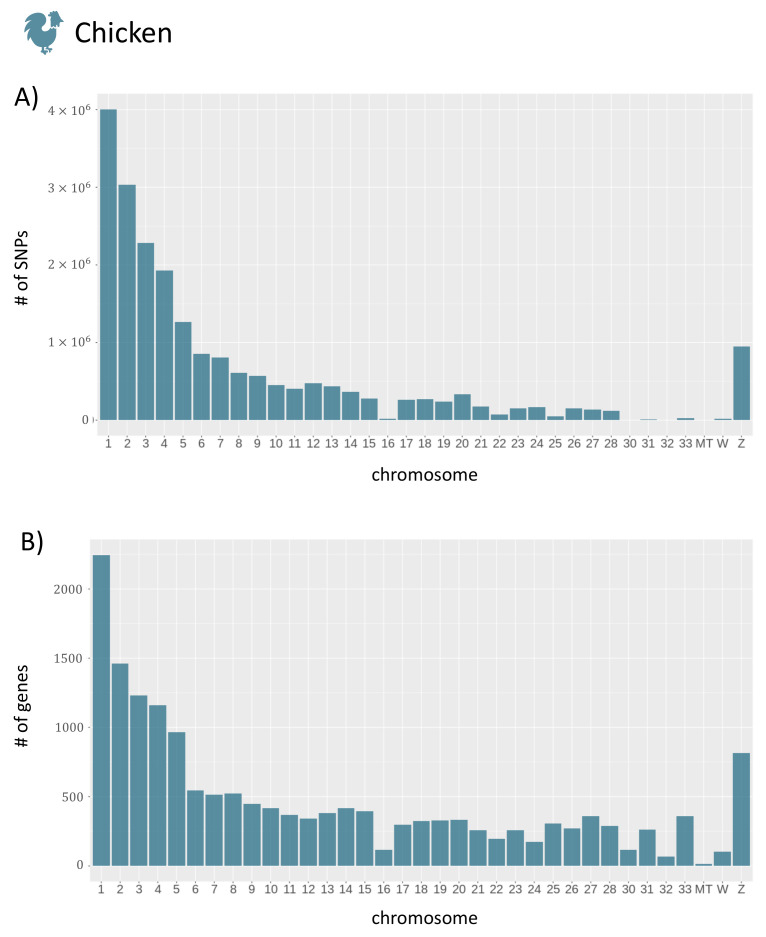
The total number of SNPs and genes for each chromosome of chicken. (**A**) The number of SNPs per chromosome. (**B**) The number of genes per chromosome. In total, 20,917,836 SNPs and 16,659 genes were reported. For plotting, the R package ggplot2 [54] was used.

To give a brief overview of the data stored in agReg-SNPdb, we show the distribution of SNPs, genes, and rSNPs in the promoter regions along the chromosomes in an exemplary manner for the species chicken. The distributions for the remaining animals can be found in Figures S2 and S3. The distributions of SNPs and genes along the chromosomes are shown in Figure 5. As expected, the number of SNPs and genes decreased largely with increasing chromosome number and, hence, with decreasing chromosome size.

**Figure 6:**
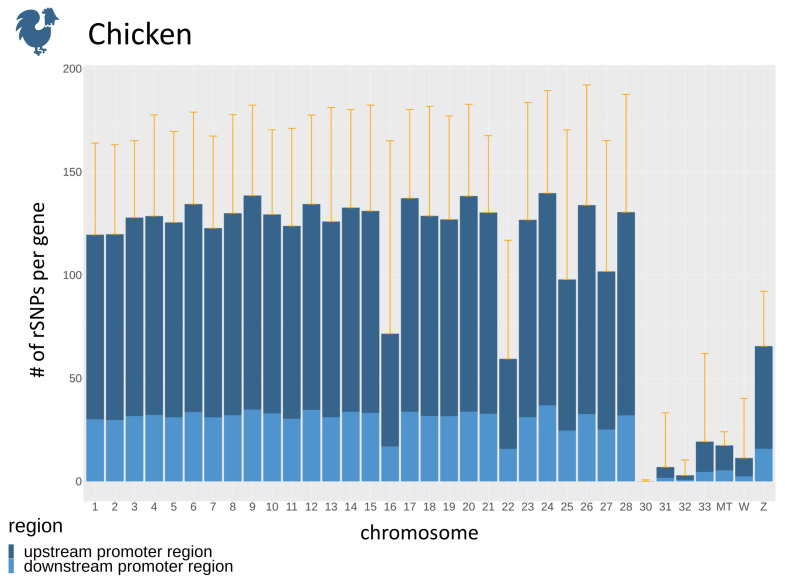
The average number of rSNPs in promoter regions per gene for each chromosome of chicken, divided into upstream and downstream promoters. The orange whiskers denote the mean plus one standard deviation.

Regarding the promoter regions, the number of SNPs in promoters is dependent on the number of genes (Figure 5B) for each chromosome. To overcome this dependency, we calculate the average number of rSNPs per gene in the upstream as well as the downstream promoter region. The average numbers of rSNPs for each chromosome in chicken revealed that most chromosomes had approximately 120 rSNPs per gene, while, on some chromosomes, only very few rSNPs per gene were found (Figure 6). Overall, by dividing the total number of rSNPs by the total number of genes, we identified on average 95.04 rSNPs within the promoter region (10 kb) of one gene in chicken.

**Figure 7:**
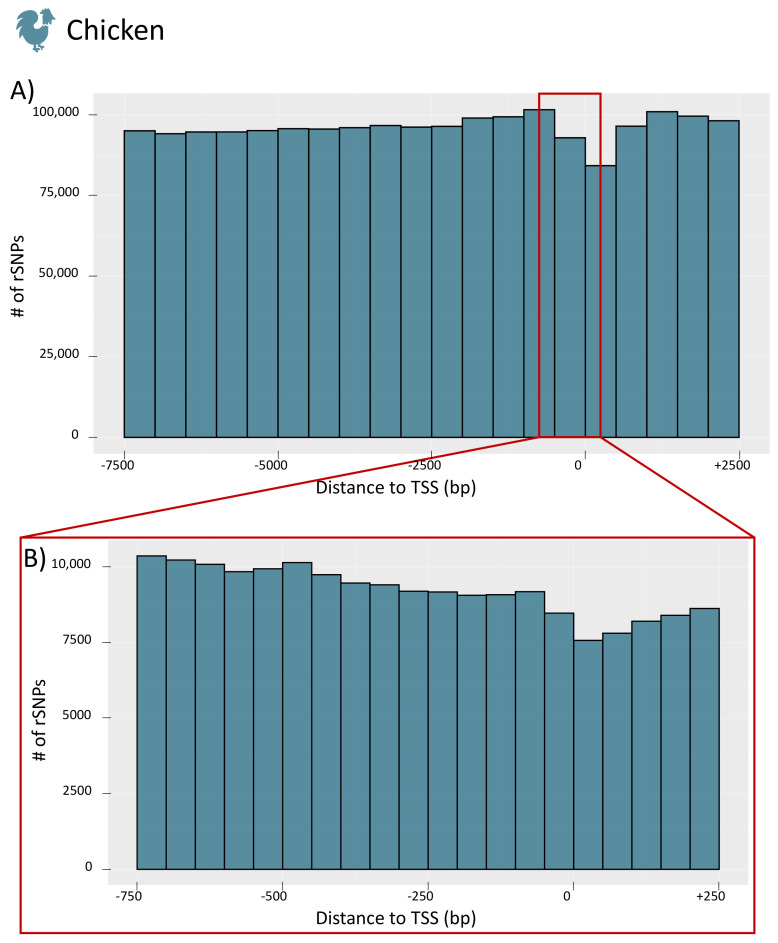
Distribution of the distances between rSNPs and the TSS of chicken. (**A**) The counts for the whole promoter region (−7.5 kb to +2.5 kb) in 500 bp intervals. The enlargement in (**B**) shows the proximal promoter region (−750 bp to +250 bp) in 50 bp intervals.

**Figure 8:**
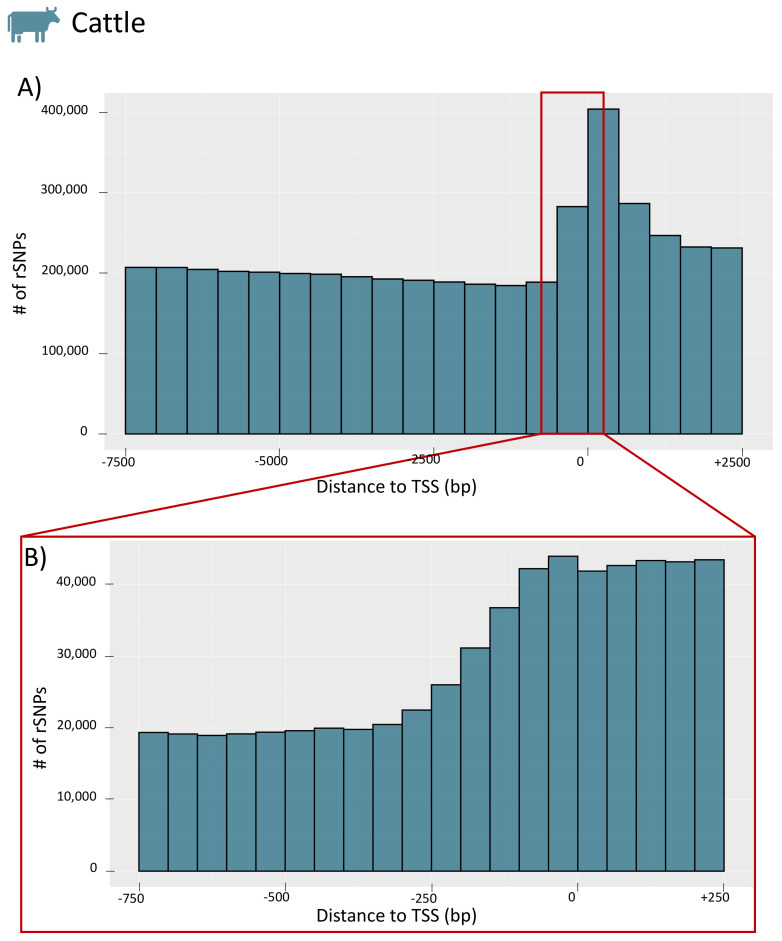
Distribution of the distances between rSNPs and the TSS of cattle. (**A**) The counts for the whole promoter region (−7.5 kb to +2.5 kb) in 500 bp intervals. The enlargement in (**B**) shows the proximal promoter region (−750 bp to +250 bp) in 50 bp intervals.

To obtain further insight into the distribution of rSNPs in the promoter regions, we investigated their genomic positions relative to the TSS for the whole promoter region (−7.5 kb to +2.5 kb) and for a smaller section (−750 bp to +250 bp) for chicken (see Figure 7A,B, respectively; the figures for the remaining species are given in Figure S4). For chicken, we observed a similar finding as in our previous study on rapeseed [33] and as previously shown in rice [53]. While there are few rSNPs in close proximity to the TSS, the number of rSNPs increases with increasing distance to the TSS. Interestingly, in cattle (as well as in dogs), we observed the opposite tendency. Many rSNPs were found around, and especially directly downstream, of the TSS, while the number decreased with the distance to the TSS (the distribution of cattle rSNPs is shown in Figure 8).

## 4. Biological Validation Based on Case-Studies

In order to validate the data stored in agReg-SNPdb, we performed literature research and assessed the importance of our findings based on selected published studies, which identified putative rSNPs that are associated with a trait under study and affect TF binding, either by prediction or as evaluated in a biological experiment.

### 4.1. Milk Protein and Fat Content in Dairy Cattle

Lum et al. [23] studied the molecular mechanism of different expression levels of the ß-Lactoglobulin (LGB) gene (also known as *MBLG* or *PAEP*), which plays an important role in the milk casein, protein, and fat content in dairy cattle. They described one rSNP in the *LGB* promoter with a G to C conversion 450 bp upstream of the TSS that was found within an activator protein-2 (AP-2) binding site. Measuring the different AP-2 binding affinities with DNase-I footprinting, they measured increased protein binding in the A promoter (G allele).

In our database, we identified the same rSNP (rs41255679, C/G), which was located in the proximal upstream promoter region of *PAEP* and caused a gain of the AP-2 binding site with the G allele (Table 4) [55]. This supports the findings of different studies reporting that AP-2 binding as well as *LGB* gene expression is enhanced by the G allele and that rs41255679 could be an important regulator of *LGB* expression [23,55,56,57].

### 4.2. Fat-Related Beef Quality Traits in Cattle

Matsumoto et al. [19] investigated the role of different bovine fat-related genes, including the gene encoding the fatty acid-binding protein 4 (FABP4). Within the *FABP4* upstream promoter, they identified two SNPs in linkage disequilibrium (*FABP4* g. −295A > G and *FABP4* g. −287A > G) that were associated with several fat-related traits, such as the carcass weight and beef marbling score. Using TFSEARCH [58], they predicted TFBSs overlapping the SNPs and altering their binding sites. In agReg-SNPdb, we identified two SNPs within the *FABP4* promoter region at a distance of 8 bp to each other and A to G conversions (respectively, T to C conversions, due to the gene’s location on the minus strand).

For the first SNP rs110055647, located 123 bp upstream of the TSS, we predicted a loss of TFBS for the Sex-Determining Region Y Protein (SRY) binding site, which is in line with the results of Matsumoto et al. [19]. For the neighboring rs109682576 (-115 bp from the TSS), we did not observe the CCAAT/enhancer-binding protein beta (cEBP/*β*) binding site predicted in their study; however, the TFBSs for Zinc finger proteins 333 (ZNF333) and 105 (ZFP105) were lost with the alternate allele, which can be seen as an extension to the results of Matsumoto et al. (Table 5) [19].

### 4.3. Chicken Egg Production

The prolactin (*PRL*) gene product is considered as an important reproductive hormone involved in diverse biological functions in vertebrates. In laying hens, it is an important regulator of egg production since an increased PRL secretion induces broodiness behaviour [28]. Liang et al. [29] examined the *PRL* 5’ promoter region and, using several populations of Chinese native Yuehuang, Taihe Silkie, and White Leghorn Layer chickens, they identified different rSNPs overlapping the predicted binding sites, including GATA-binding factor 1 (GATA-1), nuclear factor 1 (NF-1), and activator protein 1 (AP-1). Particularly for SNP rs313497646 (A/G conversion, 2048 bp upstream of the TSS), we observed the same pattern with respect to TF binding in agReg-SNPdb: only the A allele allows the binding of the NF-1 factor.

Furthermore, it has been shown that the pituitary transcription factor 1 (PIT-1) is an important activator of the *PRL* gene expression [28,29,59]. In agReg-SNPdb, we store a SNP (rs731078272, G/T), located -3086 bp from the TSS and causing a loss of the PIT-1 binding site in the T allele. This result suggests that this SNP might be an important regulator of *PRL* expression where the T variant could repress *PRL* expression, which is an important indication for further studies.

### 4.4. Fatty-Acid Composition Related Traits in Pigs

Ballester et al. [24] studied the expression of apolipoprotein (apo-) A-II (APOA2), a protein involved in the triglyceride, fatty acid, and glucose metabolisms, and identified several SNPs associated with *APOA2* gene expression and fatty acid composition traits. Four SNPs were located in the promoter region (rs322246820, rs335066625, rs339777757, and rs333406887), among which they only found one (rs333406887, C/G) influencing a predicted TFBS—in this case, a NF-1 binding site.

Similar to their result, in agReg-SNPdb, we found the SNP rs333406887 overlapping TFBSs, such as the NF-1 binding site. Furthermore, in addition to the reported change in the binding score for NF-1, we can predict several other TFBSs that are affected by this SNP. It causes, for instance, a loss of TFBS for the kruppel-like factor 6 (also called CPBP) and a gain of TFBS for zinc finger protein X-linked (ZFX) (Table 6).

## 5. Discussion

Today, it is widely known that protein–DNA interactions govern the level of gene expression in all higher organisms to a great extent. The binding of TFs to the DNA mainly occurs in the regulatory regions, such as promoters, which are found close to the transcription start of genes [60]. The effect of rSNPs on the binding of TFs has been studied extensively in single case studies in different species, and, for humans, many tools and databases exist to facilitate these analyses (see Table 1 and Table S1).

However, there is limited information available for livestock, and, to the best of our knowledge, there is no comparable data source for evaluating the effect of rSNPs. To address this lack of information, we systematically carried out a genome-wide analysis to detect rSNPs and to evaluate their consequences for TF-binding in seven animal species, which can be accessed via a web server. We showed that, by substituting a single base in a predicted TFBS, a SNP can lead to a major change in the binding affinity of the TF and, in an extreme case, even result in the disruption of the TFBS or the creation of a new TFBS.

These predictions can be of great use for scientists who have conducted: (i) an association analysis and want to reveal the underlying mechanisms caused by a SNP being significantly associated with a trait (e.g., in [19,23,33,34]); (ii) a gene expression experiment and want to identify candidate SNPs influencing the expression rate of a specific gene or a set of genes (e.g., in [24,29,33]); or (iii) a combination of both, i.e., an expression quantitative trait locus (eQTL) analysis (e.g., in [17]).

Even though our predictions are in line with many biologically tested results, as shown in the biological validation in Section 4, we note that the binding affinity of the TFs to the DNA sequence is one of the most important factors for TF binding but might not be sufficient for in vivo binding in higher organisms. Other influencing factors might include the chromatin accessibility, TF concentration, or other enhancing or repressing protein-DNA interactions, such as competitive or cooperative TF binding [3,39,61], which could not be considered in the prediction pipeline.

TF binding often occurs in a complex interplay and also includes cooperation between proximal and distal regulatory elements (promoters and enhancers) [2]. Thus, in addition to the binding of TFs in the proximal promoter regions, regulatory processes via TF-DNA interactions are also controlled by distal enhancer regions. Due to the limited knowledge of enhancer regions in livestock species, we could not incorporate these distal regulatory regions.

For our analysis pipeline, we defined a relatively wide promoter region of 7.5 kb upstream to 2.5 kb downstream of the TSS. Similarly large promoter regions were defined in previous studies ranging from 10 kb upstream to 10 kb downstream of the TSS [10,37,42,43,44,45,46,47,48] in order to overcome inaccuracies in the TSS prediction [53] and to ensure the inclusion of the biological promoter. The user has to be aware that the biological promoter region is usually smaller [53], and our website gives the opportunity to filter for smaller, user-defined promoter regions for each single gene. These considered promoter regions and the definition of rSNPs in our study (see Section 2.2.3) led to a relatively large number of rSNPs per gene—for instance, an average of 95.04 rSNPs per gene in chicken.

Interestingly, our results regarding the distribution of genome-wide rSNPs relative to the TSS showed two different patterns. In chicken, pig, sheep, horse, and goat, we observed that the region around the TSS was rather protected from sequence variations (Figure 7) as it was found in previous studies [33,53]. However, the data for cattle and dogs revealed a different picture, and we found an accumulation of SNPs and rSNPs around the TSS (Figure 8). This observation shows that the data stored in public databases, such as Ensembl, can show completely different patterns for different species, which could create biases for specific analyses.

## 6. Conclusions

To the best of our knowledge, agReg-SNPdb is the first database of regulatory SNPs for animal species of agricultural importance. It allows the users to investigate the predicted effect of an allele change on TF binding. The release of the database is an important step toward the understanding of gene regulation in the life sciences. Knowing whether a SNP causes a change in the binding affinity or even disrupts a TFBS or creates a new TFBS can be of predominant importance in order to interpret the results, from, e.g., GWAS experiments, gene expression experiments, or population studies.

The newly gained information can be used to help in genomic selection and marker establishment by identifying possibly causal rSNPs and revealing the underlying regulatory mechanisms of specific traits or diseases. Due to the regular updates of genomes as well as gene and SNP annotations, the database will be updated regularly, and, as future work, we will include several plant species with agricultural importance in agReg-SNPdb.

## Figures and Tables

**Table 1:** A summary of five recent studies that systematically investigated the effects of SNPs on regulatory elements, such as TFBSs. The analyses were done by either collecting experimentally supported and published data or by predicting the SNP impact on TF binding using prediction tools.

Name	Species	DB/Tool	Website	Characteristics	Experimentally Supported Data or Prediction
QBiC-Pred [35]	Human	Tool	http://qbic.genome.duke.edu (accessed on 16 August 2021)	TFBS prediction with regression models Prediction of changes in TF binding using ordinary least squares and evaluation of correlation between the predicted binding changes and changes in gene expression	TFBS prediction
atSNP [11] atSNP-Search [36]	Human (atSNP: organisms from Bioconductor BSGenome package [31])	Tool, DB	http://atsnp.biostat.wisc.edu (accessed on 16 August 2021)	atSNP: R package for TF binding affinity testing for rSNPs (needs a SNP and motif set as input) atSNP Search: DB for human SNP-motif pairs and the respective significance	TFBS prediction
INFERNO [37]	Human	Tool	http://inferno.lisanwanglab.org (accessed on 16 August 2021)	Inferring causal variants from genome-wide association studies (GWAS) within annotated regulatory regions as enhancers including tissue context TFBS prediction with HOMER	TFBS prediction
rSNPBASE [38], rSNPBASE 3.0 [10]	Human	DB	http://rsnp.psych.ac.cn (accessed on 16 August 2021) http://rsnp3.psych.ac.cn (accessed on 16 August 2021)	DB of rSNPs with references to regulatory elements Includes proximal and distal regulatory regions, post-transcriptional regulation, linkage disequilibrium (LD), and expression quantitative trait locus (eQTL) information rSNPBASE 3.0 includes regulatory element-target gene pairs for regulatory networks	experimentally supported regulatory elements
SNP2TFBS [39]	Human	DB	https://ccg.epfl.ch//snp2tfbs (accessed on 16 August 2021)	DB of human SNPs that affect TFBSs and the prediction of a consequence DB can be downloaded as text files or accessed via the website	TFBS prediction

**Table 2:** Assembly versions of the input data, including the reference genome, SNP catalog, and gene annotations. All files were downloaded from Ensembl (release 103).

Animal	Assembly Version	Download Date
Cattle	ARS-UCD1.2	1 March 2021
Pig	Sscrofa11.1	9 March 2021
Chicken	GRCg6a	25 February 2021
Sheep	Oar_rambouillet_v1.0	1 March 2021
Horse	EquCab3.0	1 March 2021
Goat	ARS1	1 March 2021
Dog	CanFam3.1	8 March 2021

**Table 3:** The number of records stored in the database tables *snp_info*, *gene_info*, *snp_region*, and *TFBS_results*.

	snp_Info	gene_Info	snp_Region	TFBS_Results
**Cattle**	88,109,946	21,656	9,335,814	9,074,371
**Pig**	58,145,647	20,267	4,385,724	4,432,047
**Chicken**	20,917,836	16,659	3,810,524	3,901,905
**Sheep**	50,164,898	20,359	3,216,474	3,205,279
**Horse**	20,331,427	20,499	1,585,207	1,713,395
**Goat**	31,331,447	19,658	1,987,914	2,015,588
**Dog**	4,725,021	19,960	494,691	489,292
**Total**	273,726,222	139,058	24,816,348	24,831,877

**Table 4:** Consequences of SNP rs41255679 (C/G), located upstream of the TSS of the bovine *LGB* gene. Allele 0 refers to a predicted TFBS in the reference sequence, while allele 1 stands for the alternate allele. A SNP causes a loss of TFBS if the considered TFBS (represented by a PWM) is only predicted for the reference allele. Consequently, a SNP causes a gain of TFBS if the TFBS is only predicted for the alternate allele.

SNP ID	Allele	PWM	Consequence
rs41255679	0	V$CTCF_01	Loss of TFBS
rs41255679	1	V$AP2ALPHA_03	Gain of TFBS

**Table 5:** Consequences of the SNPs rs110055647 and rs109682576 in the bovine *FABP4* upstream promoter with a T to C conversion. Allele 0 refers to a predicted TFBS in the reference sequence, while allele 1 stands for the alternate allele. A SNP causes a loss or gain of TFBS if the considered TFBS is only predicted for the reference or alternate allele, respectively. A SNP is considered to cause a score-change if the TFBS is predicted on both alleles (0,1) with a difference in the matrix similarity score computed by MATCH™.

SNP ID	Allele	PWM	Consequence
rs110055647	0,1	V$RHOX11_01	Score-Change
rs110055647	0	V$SRY_Q6	Loss of TFBS
rs109682576	0	V$ZNF333_01	Loss of TFBS
rs109682576	0	V$ZFP105_04	Loss of TFBS

**Table 6:** Consequences of the SNP rs333406887 (C/G) located -238 bp from the porcine *APOA2* TSS. Allele 0 refers to a predicted TFBS in the reference sequence, while allele 1 stands for the alternate allele. A SNP causes a loss or gain of TFBS if the considered TFBS is only predicted for the reference or alternate allele, respectively. A SNP is considered to cause a score-change if the TFBS is predicted on both alleles (0,1) with a difference in the matrix similarity score computed by MATCH™.

SNP ID	Allele	PWM	Consequence
rs333406887	0,1	V$NF1_Q6	Score-Change
rs333406887	0,1	V$AP2ALPHA_03	Score-Change
rs333406887	0	V$CPBP_Q6	Loss of TFBS
rs333406887	1	V$ZFX_01	Gain of TFBS

## Data Availability

https://azifi.tz.agrar.uni-goettingen.de/agreg-snpdb (accessed on 16 August 2021).

## Supplementary Materials

The following are available online at https://www.mdpi.com/article/10.3390/biology10080790/s1, Table S1: A comprehensive overview of recent studies that investigated the effects of SNPs on regulatory elements (extension of Table 1), Figure S2: Number of SNPs and genes per chromosomes for all species, Figure S3: The average numbers of rSNPs per gene for each chromosome for all species, Figure S4: Distribution of the distances between rSNPs and the TSS for all species.

## Abbreviations

The following abbreviations are used in this manuscript:

SNP	single nucleotide polymorphism
rSNP	regulatory SNP
TF	transcription factor
TFBS	transcription factor binding site
TSS	transcription start site
bp	base pair
eQTL	expression quantitative trait locus
LD	linkage disequilibrium
GWAS	genome-wide association study
PWM	position weight matrix
SQL	Structured Query Language
